# Disorder prediction-based construct optimization improves activity and catalytic efficiency of *Bacillus naganoensis* pullulanase

**DOI:** 10.1038/srep24574

**Published:** 2016-04-19

**Authors:** Xinye Wang, Yao Nie, Xiaoqing Mu, Yan Xu, Rong Xiao

**Affiliations:** 1 School of Biotechnology and Key Laboratory of Industrial Biotechnology, Ministry of Education, Jiangnan University, Wuxi 214122, China; 2 State Key Laboratory of Food Science and Technology, Jiangnan University, Wuxi 214122, China; 3 The 2011 Synergetic Innovation Center of Food Safety and Nutrition, Jiangnan University, Wuxi 214122, China; 4 Center for Advanced Biotechnology and Medicine, Department of Molecular Biology and Biochemistry, Rutgers University, Piscataway, NJ 08854, USA

## Abstract

Pullulanase is a well-known starch-debranching enzyme. However, the production level of pullulanase is yet low in both wide-type strains and heterologous expression systems. We predicted the disorder propensities of *Bacillus naganoensis* pullulanase (PUL) using the bioinformatics tool, Disorder Prediction Meta-Server. On the basis of disorder prediction, eight constructs, including PULΔN5, PULΔN22, PULΔN45, PULΔN64, PULΔN78 and PULΔN106 by deleting the first 5, 22, 45, 64, 78 and 106 residues from the N-terminus, and PULΔC9 and PULΔC36 by deleting the last 9 and 36 residues from the C-terminus, were cloned into the recombinant expression vector pET-28a-PelB and auto-induced in *Escherichia coli* BL21 (DE3) cells. All constructs were evaluated in production level, specific activities and kinetic parameters. Both PULΔN5 and PULΔN106 gave higher production levels of protein than the wide type and displayed increased specific activities. Kinetic studies showed that substrate affinities of the mutants were improved in various degrees and the catalytic efficiency of PULΔN5, PULΔN45, PULΔN78, PULΔN106 and PULΔC9 were enhanced. However, the truncated mutations did not change the advantageous properties of the enzyme involving optimum temperature and pH for further application. Therefore, Disorder prediction-based truncation would be helpful to efficiently improve the enzyme activity and catalytic efficiency.

Pullulanase (EC 3.2.1.41) catalyzes hydrolysis of the α-1,6 glycosidic linkages in pullulan, amylopectin, and the α- and β-limit dextrins of amylopectin^1^. During the saccharification process, glucoamylase and pullulanase act simultaneously on the substrate, and addition of pullulanase can improve the substrate conversion rate and reduce the amount of glucoamylase^2^. Thus, there is a great commercial interest of using pullulanase in industrial application, especially the production of high-glucose syrup from starch, due to the ability of pullulanase to hydrolyze the α-1,6 linkages in amylopectin.

Various pullulanases have been identified and heterologously expressed^3^,^4^,^5^. However, the expression levels of recombinant pullulanase were yet limit in reported literatures, and low enzyme activity is currently the major challenge limiting the large-scale production and widespread application of pullulanase. One approach to improve the expression level is generally by transformation and modification of the external component for gene expression, but with limited success for pullulanase^3^,^5^,^6^, foreign gene could not always be expressed efficiently in heterologous strain^5^,^7^.

In recent years, genetic modification offers an alternative option for development of new enzymes with industrial application potential. Young-Min Kim *et al.* reported that truncation of N- and C-terminal regions of *Streptococcus mutans* dextranase enhanced the catalytic activity^8^. Removal of the signal peptide significantly affected the functional expression of *Bacillus licheniformis* γ-glutamyltranspeptidase in recombinant *Escherichia coli*^9^. Truncation of N-terminal regions of *Digitalis lanata* progesterone 5β-reductase altered its catalytic efficiency and substrate preference^10^. Xiangtao Liu *et al.* found that truncation of the alternate hydrophilic and hydrophobic clusters at the N-terminus resulted in a high maleate cis–trans isomerase activity for the biocatalytic production of fumaric acid^11^. High-quality production of human α-2,6-sialyltransferase in *Pichia pastoris* required control over N-terminal truncations by host-inherent protease activities^12^. Enzyme Bio-Systems Company published in the patent that the exocytosis level of pullulanase from *B. naganoensis* was increased 1.6 times by truncated the N-terminal 106 amino acid residues^13^. From the above reported examples, histidine tag, hydrophilic and hydrophobic clusters, and domain structures were usually selected as the potential truncated regions. By truncating N- or C-terminal regions, protein of high molecular weight and complicated structure tended to achieve high secretion ratio and low tendency to form inclusion body. However, few literatures have been reported to apply disorder and secondary structure prediction tools for truncation-based construct optimization to improve expression level and enzyme activity.

In our previous work, the pullulanase from *B. naganoensis* (PUL) has been identified and the corresponding encoded gene *pul* was heterologously expressed in recombinant *E. coli*^14^. In this study, on the basis of disorder prediction of the amino acid sequence of PUL, eight truncated mutants were constructed. Then the influence of these truncations at the N- or C-terminal regions on the enzyme activity and properties was investigated.

## Results and Discussion

### Disorder prediction and construct optimization

The amino acid sequence of the PUL was analyzed using the Disorder Prediction Meta-Server (DisMeta). DisMeta analyzes a protein sequence using eight disorder prediction algorithms (DISEMBL, DISOPRED2, DISpro, FoldIndex, GlobPlot2, IUPred, RONN and VSL2). From each algorithm, disorder predictions are provided separately by the server, along with a plot showing the disorder levels of all amino acid residues in a protein sequence predicted by the algorithms^15^. In addition, protein sequence are also analyzed by seven sequence analysis tools (coils, ANCHOR, SignalP, TMHMM, SEG, PROFphd and PSIPred) to provide the information including secondary structure, binding site and complexity prediction^16^. The predicted results are presented in an easy-to-read graph that allows comparison of different algorithms simultaneously.

**Figure 1:**
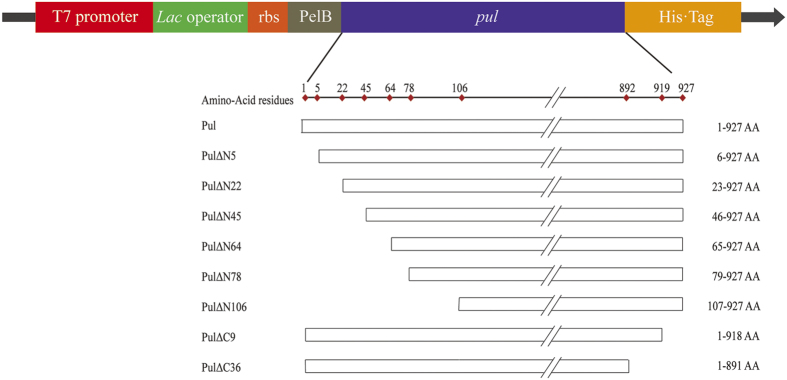
Diagrammatic sketch of the full-length PUL and its truncated mutants. The sites for truncation were determined by analyzing the amino acid sequence using both the methods of disorder prediction algorithms and sequence analysis tools. The up- and downstream sequences of the *pul* in expression plasmid were also presented, involving the expression elements in pET-28a-PelB, N-terminal PelB signal sequence and C-terminal His-tag sequence.

The N- and C-terminus of the PUL were predicted to be disordered by the majority of algorithms, whereas the conserved sequences were predicted to be ordered (Fig. S1). The results of secondary structure consensus generated from PROFsec and PSIPred indicated several β-sheet structures at both the N- and C-terminus of the enzyme, with the intervals of loop structures. It could be obviously observed that there were five distinct loops covering the residues 1–5, 13–22, 27–45, 64–78 and 99–110 at the N-terminus and two distinct loops covering the residues 918–927 and 891–904 at the C-terminus. Thus, it would be feasible to truncate the flexible loops at the corresponding sits to investigate the influence of these disordered regions on the activity and catalytic efficiency of the enzyme. In addition to the secondary structure analysis for identification of disordered loops, the graph of disorder consensus further indicated the possible disordered sites in the protein sequence. As shown in Fig. S1, with the plot showing disorder levels of involved sites in disorder consensus, the sites of much disorder in the N- and C-terminal sequences were consistent with the predicted loop structures, covering the residues 1–5, 16–22, 33–45, 74–78 and 100–106 at the N-terminus and 891–903 and 918–927 at the C-terminus. For the fragment 46–64, although its secondary structure was predicted as two β-sheets, the disorder levels of these sites were somewhat high and hence the fragment was also taken into account for truncation. Therefore, the cutting sites to truncate the disordered regions at the N- or C-terminus of the PUL were proposed as 5, 22, 45, 64, 78, 106, 891 and 918. Consequently, on the basis of the disorder prediction consensus, combined with secondary structure prediction and domain boundary identification, eight truncated mutants were constructed, including PULΔN5, PULΔN22, PULΔN45, PULΔN64, PULΔN78 and PULΔN106, by deleting the first 5, 22, 45, 64, 78 and 106 residues from the N-terminus, and PULΔC9 and PULΔC36 by deleting the last 9 and 36 residues from the C-terminus (Fig. 1).

### Activities and kinetic parameters of truncated mutants

**Figure 2:**
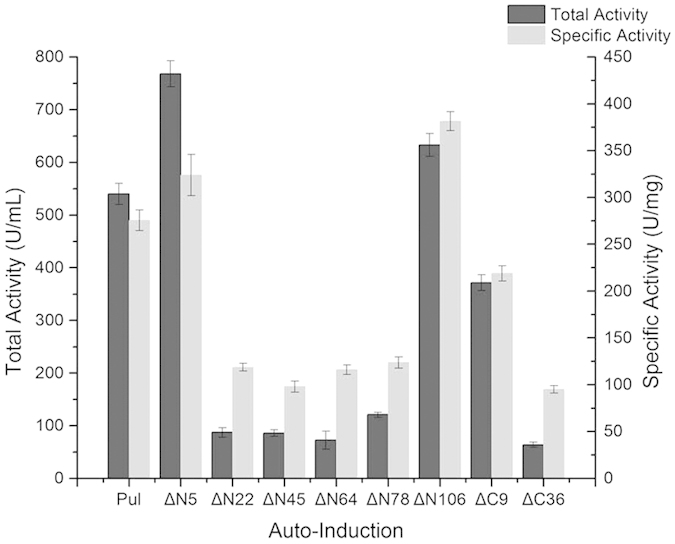
Total enzyme activities and specific activities of the PUL and its truncated mutants with auto-induction. Total activity was defined as the sum of extracellular and intracellular activity. All the values of enzymatic activities were averaged from three replicates with standard deviations, and significant differences (p < 0.05) were measured.

With the primers designed according to the results of disorder prediction and the principle of codon usage (Table S1), the genes encoding these N- or C-terminally truncated pullulanases were subcloned into the recombinant expression vector pET-28a-PelB and expressed in *E. coli* BL21 (DE3) by auto-induction. As known, heterologous protein expressed in secretory recombinant *E. coli* system is generally transported to the periplasmic space by the available signal peptide^17^. Therefore, most activities were detected in the periplasmic space but not in the supernatant. In this work, supplementation of glycine was not applied for extracellular accumulation of the pullulanase. Thus, the total pullulanase activities from both the culture supernatant and the periplasmic space were measured to evaluate the effect of truncations on the expression of the PUL. Of these mutants, PULΔN5 and PULΔN106 exhibited the total enzyme activities of 768 U mL^−1^ and 633 U mL^−1^, which were 42% and 17% higher than that of the wild-type PUL, respectively. However, other truncated mutants performed somewhat lower total pullulanase activities, compared with the wild-type PUL (Fig. 2).

**Figure 3:**
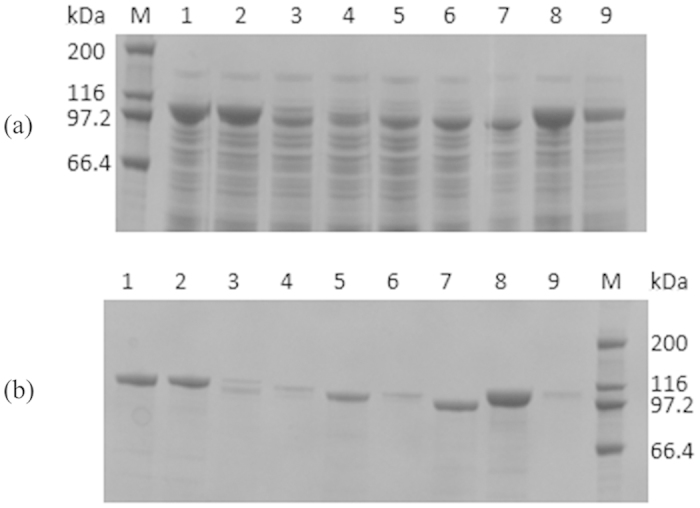
SDS-PAGE analysis of (**a**) the cell-free extracts and (**b**) the purified recombinant enzymes. For cell-free extracts, lane 1–9 meant the total soluble protein of the construct PUL, PULΔN5, PULΔN22, PULΔN45, PULΔN64, PULΔN78, PULΔN106, PULΔC9 and PULΔC36, respectively. For purified enzymes, lane 1–9 meant the purified PUL, PULΔN5, PULΔN22, PULΔN45, PULΔN78, PULΔN64, PULΔN106, PULΔC9 and PULΔC36, respectively. Lane M meant the protein molecular weight marker. The amount of loaded sample was 10 μL in each lane.

To understand the possible reason leading to the difference of total pullulanase activities among the truncated and full-length enzymes, SDS-PAGE analysis of the cell-free extracts after expression was conducted for the mutants and the wild-type enzyme. The results indicated that all the truncated proteins could be expressed as soluble form and compared with the wild-type PUL, high-level expression of target protein was obviously achieved for the mutants of PULΔN5 and PULΔC9 (Fig. 3a). Additionally, the recombinant proteins were purified to homogeneity (Fig. 3b), and then the specific activities of the purified enzymes were measured. Of the truncated mutants, activities in the cells of PULΔN5 and PULΔN106 were 573 U mL^−1^ and 519 U·mL^−1^ with the amounts of purified enzymes were 1.77 mg mL^−1^ and 1.36 mgmL^−1^, respectively. Thus, PULΔN5 and PULΔN106 performed their specific activities as 324 U mg^−1^ and 382 U mg^−1^, which were 1.18- and 1.38-fold increase of the PUL, respectively (Fig. 2). Associating with the results from SDS-PAGE analysis of the expressed proteins of the constructs, the increased total activities of the mutants would be mainly caused by the improvement of the specific activity of the corresponding mutants. Therefore, truncation of the disordered region of the enzyme would be helpful to make the protein more active in catalyzing hydrolysis reaction, involving the acting behavior of binding substrate and molecular interactions. Taken together, it was worth to note that truncation of the first five amino acid residues at N-terminus significantly improved both the expression level and the enzymatic activity of the pullulanase.

To further evaluate the effect of truncation at N- or C-terminus on substrate affinity and catalytic efficiency of the enzyme, kinetic analysis of the truncated enzymes was conducted with pullulan as the substrate at 60 °C and pH 4.5. As shown in Table 1, all of the truncated mutants exhibited lower *K*_m_ values than the wild-type enzyme, indicating that deletion of the disordered region of the enzyme would increase the affinity of the enzyme towards the tested substrate. On the other hand, the *k*_cat_*/K*_m_ values of PULΔN5, PULΔN45, PULΔN78, PULΔN106 and PULΔC9 were higher than that of the wild-type enzyme, suggesting that enhanced catalytic efficiency was achieved for the enzyme. Of the truncated mutants, PULΔN5 gave the highest catalytic efficiency from the kinetic parameters, with the *k*_cat_*/K*_m_ value of 2.3-fold increase of the wide-type enzyme. Thus, disorder prediction-based construct optimization would improve the activity and catalytic efficiency of the pullulanase.

### Structural analysis of influence of truncations on enzyme function

**Figure 4:**
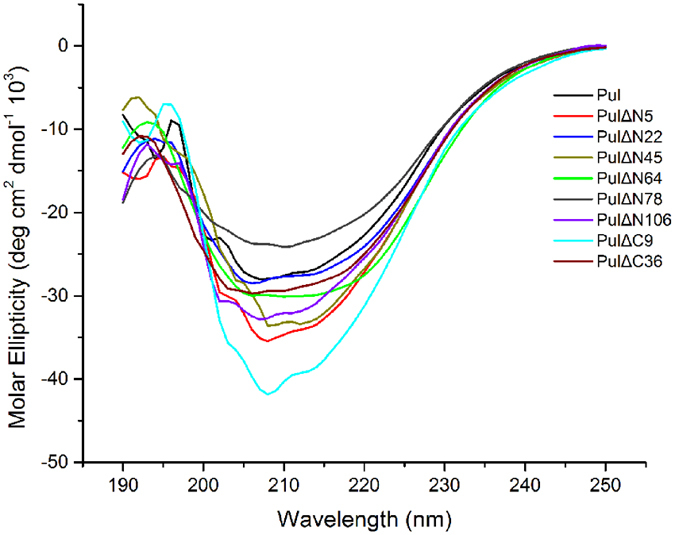
Circular dichroism analysis of the secondary structures of the wild-type PUL and the truncated mutants. The far-UV spectra of the wild-type PUL and the truncated mutants were recorded at 20 °C.

In this study, specific activity, substrate affinity and catalytic efficiency of the PUL were improved by disorder prediction-based truncation at the N- or C-terminus of the enzyme. To investigate the effects of these truncations on the structural elements, especially the composition of secondary structures, circular dichroism spectrum was applied to analyze the change of secondary structures of the truncated mutants, compared with the wild-type enzyme. As shown in Fig. 4, most of the truncated mutants performed slight difference from the PUL in the circular dichroism spectrum and the composition of secondary structures. Associating with the kinetic parameters of the mutants, change of secondary structures, especially the content of unordered loop, generated from truncation of disordered region would cause significant changes of *k*_cat_ and *V*_max_ values, while the mutants with the percentage of unordered loop close to that of the wild-type enzyme, such as PULΔN5 and PULΔN106, would be more favorable for the catalytic activity of the enzyme (Table S2).

From the fact that truncated mutation improved the specific activity and catalytic efficiency of the enzyme, especially for the mutants of PULΔN5 and PULΔN106 from N-terminus truncation, influence of truncation on the function of the enzyme was further analyzed from the respect of structure information. By homology searching of the amino acid sequence of the PUL in NCBI using the database of PDB, the pullulanase from *B. acidopullulyticus* (*Ba*Pul13A) (PDB ID: 2WAN) performed the highest identity (64%) to the PUL. However, the protein structure of *Ba*Pul13A indicated that the N-terminal domain (CBM41) was disordered and the domain structure could not be solved entirely^17^. The pullulanase from *Klebsiella pneumoniae* (low identity of 28% to PUL) was also reported to possess an N-terminal domain (N1) with the highest average B-factor, meaning high flexibility of the domain structure^18^. Although it would not be feasible to have a view at the whole structure of PUL by homology modeling, it was presumed that the N-terminal domain of PUL was also disordered, corresponding to the results from sequence analysis.

**Figure 5:**
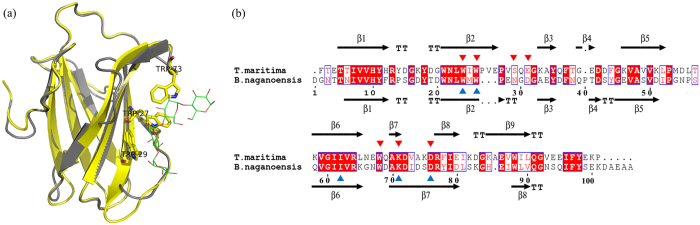
Comparison between the N-terminal domain of PUL and *Tm*CBM41 from structure and sequence analysis. (**a**) Structure overlap of the N-terminal domain of PUL (grey) and *Tm*CBM41 (yellow) with carbohydrate-binding sites in stick and the ligand in line. (**b**) Amino acid sequence alignment of the N-terminal domain of PUL and *Tm*CBM41 with the secondary structures, respectively. The carbohydrate-binding sites were indicated above and below the sequences with red and blue triangles for *Tm*CBM41 and PUL, respectively.

As known, 3D structure of pullulanase generally consists of several domain structures, e.g. CBM41-X45a-X25-X45b-CBM48-GH13 in *Ba*Pul13A, N1-N2-N3-A-C in the *K. pneumoniae* pullulanase and N1-N2-A-C in the pullulanase from *Anoxybacillus* sp. (PulA)^18^,^19^,^20^. The N-terminal domain CBM41 or N1 has been identified as the carbohydrate-binding module, interacting with oligosaccharide molecules. Functioning like a lid, the N-terminal domain had a conformational change for substrate accommodation and played an important role in assisting substrate binding for catalytic activity^20^. On the basis of the knowledge on function of the N-terminal domain of known pullulanases, the N-terminal sequence of PUL (residues 1–110) was analyzed by homology searching in NCBI using the database of PDB. The searching result revealed that the N-terminal sequence of PUL was homologous to the conserved domain of CBM41 pullulanase superfamily (Fig. S2). Additionally, a family 41 carbohydrate-binding module from *Thermotoga maritima* pullulanase (*Tm*CBM41) (PDB ID: 2J71) was found to perform the highest identity (45%) to the N-terminal sequence of PUL. Then the structure of the N-terminal domain of PUL was predicted by homology modeling using *Tm*CBM41 structure as the template. As shown in Fig. 5, the backbone structures of the N-terminal domain of PUL and *Tm*CBM41 matched in the structure alignment and the key residues for ligand binding, e.g. Trp, Lys and Asp recognizing the α-D-glucosyl-maltotriose unit in pullulan, were conserved in the N-terminal domain of PUL^21^. Furthermore, the architectures of the active sites of *Tm*CBM41 were structurally well conserved with the N-terminal domain (N1 domain) of the *K. pneumoniae* pullulanase, for which the structure-function relationship has been clarified by solving the complex structures of the enzyme^19^,^21^. Therefore, the N-terminal domain of PUL comprising obvious disordered regions would be involved in substrate recognition, and substrate binding would cause significant conformational change of the domain to accommodate substrate.

It was presumed that truncation of the PUL, especially at the N-terminus, would diminish the function of the domain as an entire lid and lead to the approach of substrate to the active center more easily, resulting in decreased *K*_m_ values of the truncated mutants. However, as disruption of domain structure by truncation, the mutants, including PULΔN22, PULΔN45, PULΔN64, PULΔN78, PULΔC9 and PULΔC36, exhibited declined specific activities and *k*_cat_ values. Because the conserved CBM41 domain mainly covered the residues 6–100 of the N-terminal sequence of PUL (Fig. S2), truncation of the first disordered loop (residues 1–5) at N-terminus (PULΔN5) would be helpful to stabilize the domain structure and improve its catalytic activity. Nevertheless, truncation of the whole N-terminal domain (PULΔN106) would not structurally affect the catalytic domain of the enzyme^22^, and hence did not have much influence on its *k*_cat_ and *V*_max_ values, while enhanced the total activity from heterologous expression. Therefore, Compared with the wild-type enzyme, the enhanced substrate affinity and catalytic efficiency indicated that the truncation of disordered region of the enzyme would make it more active and enhance its ability of binding and interacting with the substrate of mixed α-1,4/α-1,6 linked D-glucan polysaccharides such as pullulan, which was consistent with the case of the pullulanase from *B. acidopullulyticus* from the viewpoint of structure-function relationship^18^.

### Effects of pH and temperature on the activities of truncated mutants

**Figure 6:**
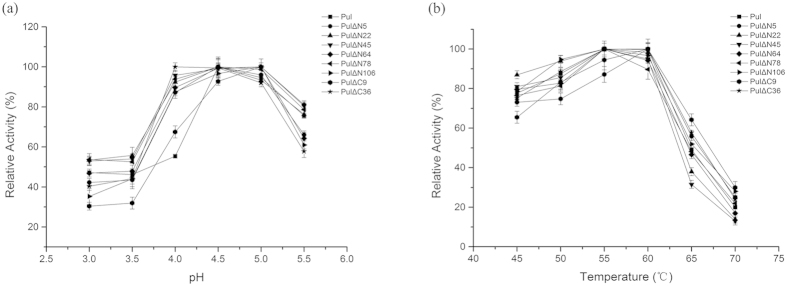
Effects of (**a**) pH value and (**b**) temperature on the enzyme activities of the PUL and its truncated mutants. Activity was measured in 0.1 M sodium acetate buffer from pH 3.0 to pH 5.5 or at the temperatures ranging from 45–70 °C. The highest activity was taken as 100%. The error bars showed the standard deviations of three replicates.

Molecular modification by mutation or truncation would generally introduce the change of structure and function of the enzyme. For the truncated mutants involving deletion at N- or C-terminus, the effects of pH value and temperature on the enzyme activity were investigated for these mutants, respectively. As shown in Fig. 6, for the activity and catalytic efficiency-increased mutants, such as PULΔN5 and PULΔN106, the values of optimal pH and temperature were 4.5 and 60 °C, respectively, indicating that these truncations did not change the pH and temperature profile of the pullulanase. According to the requirements of saccharification process involving pullulanase, the enzyme properties of optimal pH and temperature fit well the working conditions. Therefore, with respect to the application potential, the positive candidates with enhanced enzyme activity and catalytic efficiency were successfully achieved by truncation of the disordered regions of the PUL.

**Figure 7:**
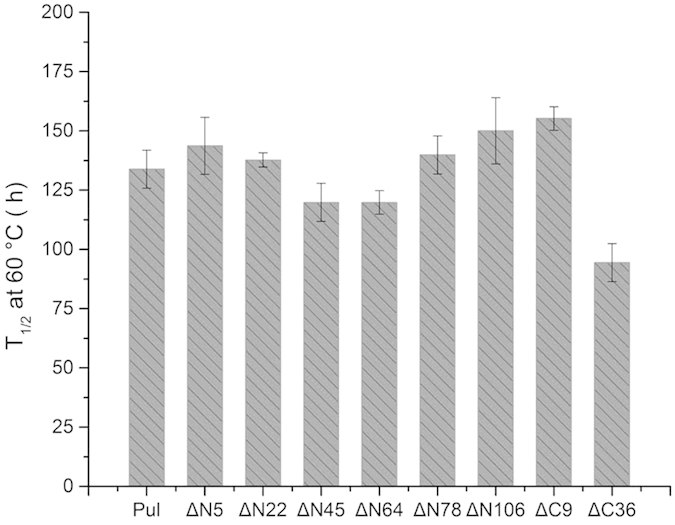
The half-lives of the PUL and its truncated mutants at 60 °C. The error bars showed the standard deviations of three replicates.

In addition to the features of optimal pH and temperature, stability as another important property of pullulanase was further evaluated towards the mutants derived from disorder prediction-based construct optimization. The residual activities of the truncated mutants and the wild-type enzyme were measured after incubation at 60 °C and pH 4.5 for different periods of time. As shown in Fig. 7, PULΔN5, PULΔN78, PULΔN106 and PULΔC9 of the mutants performed higher stability than the PUL. Compared with the wild-type enzyme, the half-lives (T_1/2_) were increased to 143.7, 139.8, 150.0 and 155.2 h for the positive mutants of PULΔN5, PULΔN78, PULΔN106 and PULΔC9, respectively. Correspondingly, the half-life of the enzyme was prolonged to 1.16 folds improvement of the original level (133.8 h). This finding was consistent with the observation that deletion or replacement of unstable domains or peptides would enhance the stability of proteins^23^,^24^,^25^,^26^. Therefore, with the unchanged features suitable for saccharification process, the pullulanase mutants, such as PULΔN5, were created to be more active and stable by disorder prediction-based construct optimization and it would be more feasible to use the developed enzyme in potential applications.

## Conclusion

On the basis of bioinformatics analysis concerning disorder and secondary structure prediction, construct optimization by truncation of disordered regions at the N- or C-terminus of the pullulanase PUL was carried out and eight truncated mutants were created consequently. Compared with the wild-type enzyme, the mutants with increased total activity and specific activity, such as PULΔN5 and PULΔN106, were achieved by truncation of the N-terminal disordered peptides. Because the expression levels of the mutants were not much higher than that of the wild-type enzyme, improvement of specific activity by truncation of disordered region would be the main reason for the increased total activity of the expressed enzyme. Further investigation on the difference of kinetic parameters between the mutants and the wild type indicated that deletion of the disordered region would increase the enzyme affinity towards the involved substrate and also its catalytic efficiency. Additionally, truncation of disordered regions of the enzyme did not give negative effects on its catalytic properties involving optimum pH and temperature and stability. In this work, the positive mutant PULΔN5 was consequently achieved with higher specific activity and catalytic efficiency, which would be promising for further industrial application. Therefore, disorder prediction-based construct optimization efficiently improved the activity and catalytic efficiency of the pullulanase, and would be considered as an efficient approach to enhance activity of desired enzymes. Our results provided experimental evidence that truncation of the disordered regions influenced the structure and function of the enzyme in some level, though understanding how different disordered states associate with the enzyme function will require more study.

## Methods

### Materials and strains

The strains including *E. coli* JM109 and *E. coli* BL21 (DE3) were used as the recombinant hosts for the gene cloning and expression, respectively. The recombinant plasmid pET-28a-PelB-*pul* bearing the pullulanase-encoded gene *pul* (GenBank Accession No. JN872757) from *B. naganoensis* was constructed as described in the previous work^14^. The polysaccharide of pullulan for determination of pullulanase activity was purchased from Tokyo Kasei Kogyo Co., Ltd (Japan). Restriction endonucleases, PrimerStar HS DNA polymerase and ligase were obtained from TaKaRa Biotechnology Co., Ltd (Dalian, China). Bradford Protein Assay Kit was purchased from TIANGEN Biotech Co., Ltd (Beijing, China). The DNA primers were synthesized by Sangon Co., Ltd (Shanghai, China) and Plasmid Mini Kit was obtained from Omega bio-tek Co., Ltd (Norcross, GA, USA). All other materials were of analytical grade and commercially available.

### Disorder prediction with DisMeta server

The DisMeta server (www-nmr.cabm.rutgers.edu/bioinformatics/disorder) employs a wide range of disorder prediction tools and several sequence-based structural prediction tools. The amino acid sequence of the pullulanase PUL was analyzed using DisMeta.

### Construction of truncated mutants

The primers in Table S1 for truncation were designed according to the principle of codon usage. The mutations were generated by PCR using PrimeSTAR HS DNA polymerase with the recombinant plasmid pET-28a-PelB-*pul* as the template. Each reaction vessel in a final volume of 50 μL contained 25 μL PrimerStar HS DNA polymerase, 0.5 μL forward primer and 0.5 μL reverse primer, and 1 μL DNA template. After DNA amplification (initial denaturation at 98 °C for 3 min; 30 cycles: denaturation at 98 °C for 45 s, annealing at 58 °C for 30 s, elongation at 72 °C for 180 s; final elongation at 72 °C for 10 min), the PCR products were analyzed by agarose gel electrophoresis and DNA sequencing^14^,^27^.

The PCR products and the plasmid pET-28a-PelB were both digested by the restriction endonucleases at the sites of *Xho*I and *Bam*HI and then ligated to obtain the recombined plasmids. The plasmids containing correct mutated genes confirmed by sequencing were finally transformed into *E. coli* BL21 (DE3) for expression of the mutants of the pullulanase.

### Expression and purification of recombinant proteins

A modified auto-induction medium^28^,^29^, containing 10 g·L^−1^ β-lactose, 1.0 g·L^−1^ glucose, 50 g·L^−1^ glycerol, 6.8 g·L^−1^ KH_2_PO_4_, 0.25 g·L^−1^ MgSO_4_, 10 g·L^−1^ tryptone, 5 g·L^−1^ yeast extract, 7.1 g·L^−1^ Na_2_HPO_4_, 0.71 g·L^−1^ Na_2_SO_4_ and 2.67 g·L^−1^ NH_4_Cl, with the pH value adjusted to 7.5, was used for high-level production of the pullulanase and the mutants. For protein expression in auto-induction medium, *E. coli* BL21(DE3) harboring recombinant plasmid was inoculated into 5 mL LB medium in the presence of kanamycin (50 μg·mL^−1^) and incubated at 37 °C and 200 rpm for 10 h. Then the *E. coli* culture of 1 mL was transferred into a 250 mL flask containing 50 mL auto-induction medium supplemented with kanamycin (50 μg·mL^−1^). After cultivation at 37 °C and 200 rpm for the first 2 h, the culture was incubated at 20 °C and 200 rpm for another 60 h to produce the target protein^14^.

As heterologous protein expressed in secretory recombinant *E. coli* system is generally transported to the periplasmic space by the available signal peptide^17^, most pullulanase activities were detected in the periplasmic space but not in the supernatant, and thus the target proteins were purified from the recombinant *E. coli* cells. The cultivated recombinant *E. coli* cells were harvested by centrifugation and suspended in binding buffer (40 mM imidazole, 0.3 M NaCl, 20 mM Tris-HCl, pH 6.5) and disrupted on ice with an ultrasonic oscillator (VCX750, Sonic). The supernatant of the cell lysate, as the crude enzyme, was collected by centrifugation at 26,000 × g for 40 min at 4 °C and purified by an AKTAxpress system using HisTrap HP affinity column (GE Healthcare, USA). Elution was carried out with 500 mM imidazole in the same buffer at a flow rate of 2.0 mL·min^−1^. Then the purified fractions were exchanged into low salt buffer (10 mM Tris-HCl, pH 6.5, 0.1 M NaCl, 0.02% NaN_3_, 5 mM D,L-dithiothreitol) using disposable PD-10 desalting columns (GE Healthcare, USA)^30^.

The molecular weight and the amount of the recombinant enzyme were estimated by 10% (w/v) sodium dodecyl sulfated-polyacrylamide gel electrophoresis (SDS-PAGE). Gels were stained with Coomassie Brilliant Blue R250 and molecular weight marker (TaKaRa Biotechnology Co., Ltd., Dalian, China) with the size ranging from 10 to 200 kDa was used as the protein standard.

### Pullulanase activity assay

Pullulanase activity was assayed by measuring the aldehyde groups released during enzymatic reaction from a mixture consisting of pullulan solution and the diluted enzyme sample^31^,^32^. The reaction mixture, containing 0.2 mL 2% (w/v) pullulan in 0.1 M sodium acetate buffer (pH 4.5) and 0.2 mL enzyme solution diluted with 0.1 M sodium acetate buffer (pH 4.5), was incubated at 60 °C for 20 min. Then, the amount of released aldehyde groups was assayed using dinitrosalicylic acid (DNS) method by measuring the absorbance at 540 nm spectrophotometrically^14^. One unit of pullulanase activity was defined as the amount of pullulanase that releases 1 μmol of aldehyde groups per min under the reaction conditions^14^. Total activity was defined as the sum of extracellular and intracellular activity. Protein concentration was determined by Bradford protein assay kit (TaKaRa Biotechnology Co., Ltd., Dalian, China). All the values of enzymatic activities were averaged from three replicates with standard deviations, and significant differences (p < 0.05) were measured.

### Determination of kinetic parameters

The kinetic parameters (*K*_m_, *V*_max_ and *k*_cat_) of the pullulanase and the mutants were determined based on the method described previously^33^, where different concentrations of pullulan ranging from 0.1 to 10.0 mg·mL^−1^ were adopted. The values of *V*_max_ and *K*_m_ were obtained by fitting the initial rate data to the Michaelis-Menten equation using nonlinear regression with GraphPad Prism software^22^.

### Characterization of truncated pullulanases

The optimal pH for each recombinant enzyme was determined by measuring enzyme activity from pH 3.0 to pH 5.5 using 0.1 M sodium acetate buffer at 60 °C. The optimal temperature for each truncated enzyme was determined by measuring activity at temperatures ranging from 45–70 °C in 0.1 M sodium acetate buffer (pH 4.5). The reactions were performed with 2% pullulan as the substrate.

The enzyme stability was evaluated by measuring the half-life (T_1/2_) at 60 °C. Enzyme solution with protein concentration of 1.0 mg mL^−1^ was incubated in 0.1 M sodium acetate buffer (pH 4.5) at 60 °C. At different time intervals, the residual activities of the samples were assayed and the values of T_1/2_ at 60 °C were calculated as the method described previously^34^.

### Circular dichroism measurements

Circular dichroism spectra were obtained using a BioLogic MOS450 spectropolarimeter (Claix, France). Protein samples (0.15 mg·mL^−1^) of 200 μL in the appropriate buffer were used for measurement in quartz cuvette of 1 mm optical length. The spectra were collected at 20 °C over a range from 190–250 nm with 2 s response time, 0.1 cm path length and a 2 nm bandwidth. The scanning speed was 30 nm/min and the data pitch was set at 1 nm.

### Homology searching and modeling

Homology searching of the whole sequence or the N-terminal sequence (residues 1–110) of the PUL was carried out using NCBI protein blast tool (http://blast.ncbi.nlm.nih.gov/Blast.cgi) with the database of PDB. On the basis of sequence identity of 45% between the CBM41 domain from *T. Maritima* pullulanase (*Tm*CBM41) and the N-terminal sequence of PUL, the protein structure of *Tm*CBM41 (PDB ID: 2J71) was used as the template for homology modeling to generate the model structure of the N-terminal domain of PUL with SWISS-MODEL.

## Additional Information

**How to cite this article**: Wang, X. *et al.* Disorder prediction-based construct optimization improves activity and catalytic efficiency of *Bacillus naganoensis* pullulanase. *Sci. Rep.* **6**, 24574; doi: 10.1038/srep24574 (2016).

## Supplementary Material

Supplementary Information

## Figures and Tables

**Table 1:** Kinetic parameters of the wild-type PUL and the truncated mutants.

Enzyme	*K*_m_(mg·mL^−1^)	*k*_cat_ (s^−1^)	*V*_max_ (μmol·mg^−1^min^−1^)	*k*_cat_/*K*_m_(mL·mg^−1^s^−1^)
PUL	6.07 ± 0.92	792.79 ± 43.50	468.13 ± 27.43	130.61 ± 24.31
PULΔN5	2.88 ± 0.61	854.09 ± 42.59	507.60 ± 25.29	296.56 ± 21.45
PULΔN22	1.46 ± 0.14	243.77 ± 4.18	147.90 ± 2.53	166.96 ± 7.94
PULΔN45	0.42 ± 0.03	202.70 ± 15.85	126.40 ± 10.72	482.61 ± 25.52
PULΔN64	3.71 ± 0.43	328.26 ± 23.74	208.90 ± 19.03	88.48 ± 10.22
PULΔN78	0.70 ± 0.01	230.47 ± 13.45	149.30 ± 8.78	329.24 ± 13.98
PULΔN106	4.33 ± 0.38	757.36 ± 38.13	507.80 ± 30.87	174.91 ± 21.39
PULΔC9	3.14 ± 0.32	598.07 ± 33.25	357.00 ± 10.09	190.47 ± 25.51
PULΔC36	2.20 ± 0.01	183.42 ± 2.27	112.50 ± 1.39	83.37 ± 0.77

The values were obtained by fitting the initial rate data to the Michaelis-Menten equation using nonlinear regression with GraphPad Prism software. Values are means ± standard deviations.
