# Supplementary material for: Disorder prediction-based construct optimization improves activity and catalytic efficiency of Bacillus naganoensis pullulanase
Source: Sci Rep. 2016 Apr 19;6:24574. doi: 10.1038/srep24574 (PMC4835747; doi:10.1038/srep24574)
Supplement: Supplementary Information [file srep24574-s1.pdf]

Supplementary Information

Disorder prediction-based construct optimization improves activity and catalytic efficiency of *Bacillus naganoensis* pullulanase

Xinye Wang[1], Yao Nie[1], Xiaoqing Mu[1], Yan Xu[1,2,3] &Rong Xiao[4]

[1]School of Biotechnology and Key Laboratory of Industrial Biotechnology, Ministry of Education, Jiangnan University, Wuxi214122, China.

[2]State Key Laboratory of Food Science and Technology, Jiangnan University, Wuxi 214122, China.

[3]The 2011 Synergetic Innovation Center of Food Safety and Nutrition, Jiangnan University, Wuxi 214122, China.

[4]Center for Advanced Biotechnology and Medicine, Department of Molecular Biology and Biochemistry, Rutgers University, Piscataway, NJ 08854, USA.

Correspondence and requests for materials should be addressed to Y.N. (email: ynie@jiangnan.edu.cn) and Y.X. (email: yxu@jiangnan.edu.cn).

Disorder prediction with DisMeta server

The overall process began when the amino acid sequence of the pullulanase PUL was analyzed by a bioinformatics-based tool evaluating the protein sequence for regions those are likely to be disordered. The Northeast Structural Genomics Consortium (NESG) has developed a web-based tool (DisMeta) to aid high throughput construct design. The DisMeta server (www-nmr.cabm.rutgers.edu/bioinformatics/disorder) employs a wide range of disorder prediction tools and several sequence-based structural prediction tools[1].

When approaching the pullulanase structure, the mission of the DisMeta server was to identify stable folded regions of the protein and to gain functional insights based on bioinformatics analysis.

Homology searching

Homology searching of the whole sequence or the N-terminal sequence (residues 1-110) of the PUL was carried out using NCBI protein blast tool (http://blast.ncbi.nlm.nih.gov/Blast.cgi) with the database of PDB.

Circular dichroism measurements

Circular dichroism spectra were obtained using a BioLogic MOS450 spectropolarimeter (Claix, France). Protein samples (0.15 mg/mL) of 200 μL in the appropriate buffer were used for measurement in quartz cuvette of 1 mm optical length. The spectra were collected at 20°C over a range from 190 to 250 nm with 2 s response time, 0.1 cm path length and a 2 nm bandwidth. The scanning speed was 30 nm/min and the data pitch was set at1 nm.

To calculate the secondary structure fractions of the wide-type PUL and the truncated mutants, the DICHROWEB web server (http://dichroweb.cryst.bbk.ac.uk) was used to

analyze circular dichroism spectroscopic data and provide calculated secondary structure contents. SELCON3 and Set 7 (190-240 nm) were selected as the analysis algorithms and reference database, respectively.

Table S1. The oligonucleotides used for PCR to obtain the truncated genes of *B. naganoensis* pullulanase (PUL). The underlined sequences represented the restriction endonuclease sites.

Name	*Bam*HI-forward primer	*Xho*I-reverse primer
PULΔN5	5’-CGCGTGGATCCAAACATCGTAGTCC-3’	5’-ATTCCCTCGAGTTTACCATCAGATGGGCT-3’
PULΔN22	5’-GAACAGGATCCACTTTGGATGTGGCC-3’	5’-ATTCCCTCGAGTTTACCATCAGATGGGCT-3’
PULΔN45	5’-GTTCAGGATCCAGAGGTTGCAAGTGTGG-3’	5’-ATTCCCTCGAGTTTACCATCAGATGGGCT-3’
PULΔN64	5’-GAACAGGATCCAAAAGGAAATTGGGATGC-3’	5’-ATTCCCTCGAGTTTACCATCAGATGGGCT-3’
PULΔN78	5’ -GCGCCCGGATCCAATCGATTTAAG-3’	5’-ATTCCCTCGAGTTTACCATCAGATGGGCT-3’
PULΔN106	5’-GTCGAGGATCCAGCACAACCTGCTGTAAG-3’	5’-ATTCCCTCGAGTTTACCATCAGATGGGCT-3’
PULΔC9	5’-GAACAGGATCCAGATGGGAACACCACAAAC-3’	5’-GCGCGCTCGAGATGAAGAATCATCATAGATA-3’
PULΔC36	5’-GAACAGGATCCAGATGGGAACACCACAAAC-3’	5’-ATTCACTCGAGCGTCGCATTGATTTCC-3’

Table S2. Calculated secondary structure fractions of the wide-type PUL and the truncated mutants.

Enzyme	Helix1 (%)	Helix2 (%)	Strand1 (%)	Strand2 (%)	Turns (%)	Unordered (%)	Total (%)
PUL	6.9	13.8	17.2	6.9	19.0	36.2	100.0
PULΔN5	6.9	13.8	17.3	7.0	19.3	36.9	101.1
PULΔN22	7.0	13.6	17.2	7.1	19.2	40.0	104.0
PULΔN45	20.9	13.6	4.6	6.9	23.6	31.9	101.5
PULΔN64	6.9	13.6	17.2	7.1	19.1	38.9	102.8
PULΔN78	7.0	13.8	17.2	7.0	19.1	37.4	101.4
PULΔN106	7.0	13.8	17.4	7.1	19.4	36.9	101.5
PULΔC9	20.9	13.5	4.8	6.9	23.6	31.8	101.6
PULΔC36	7.0	13.7	17.4	7.2	19.3	38.5	103.1

Figure S1. Disorder region and secondary structure prediction by the Disorder Prediction Meta-Server (DisMeta).

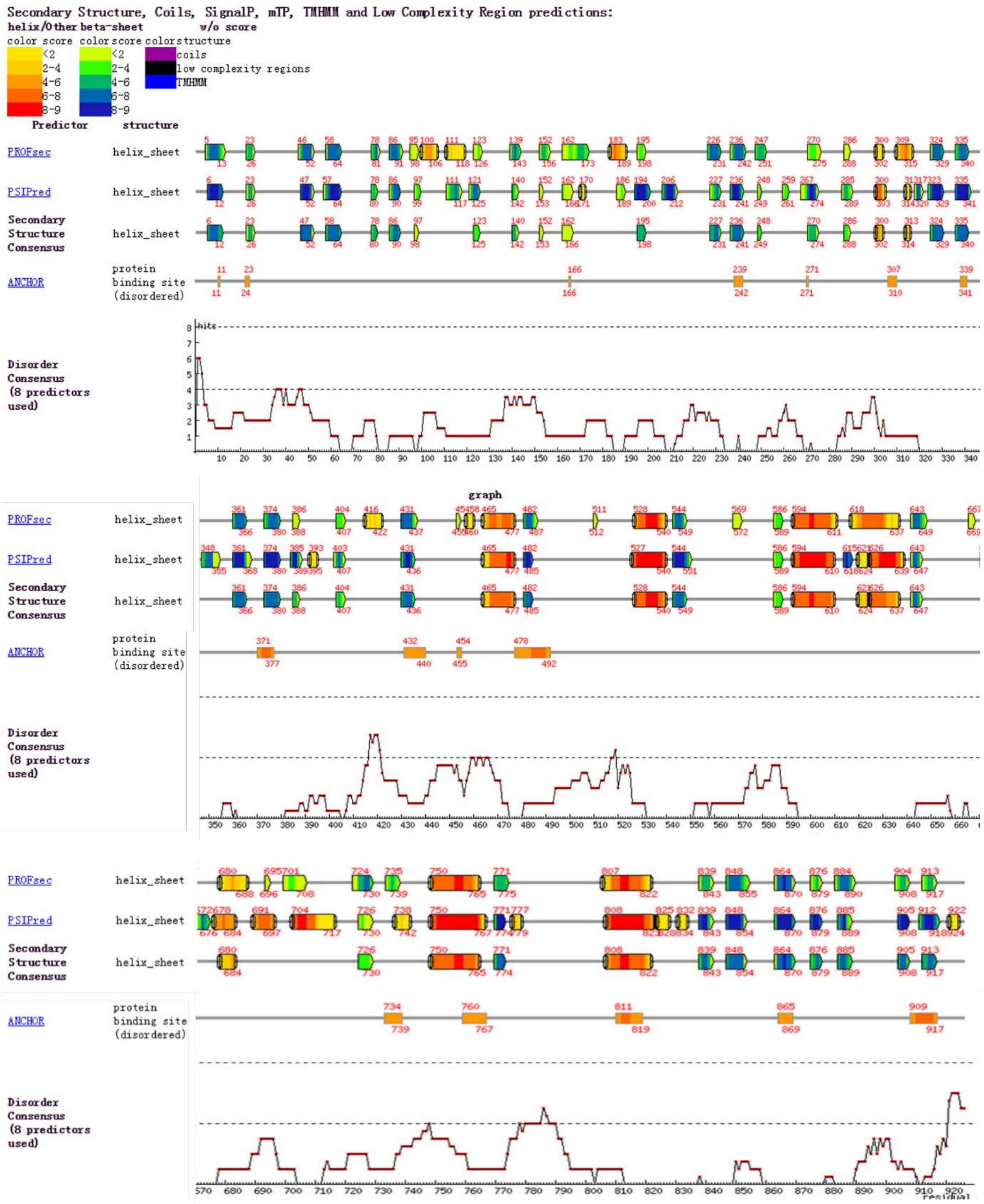

Figure S2. Homology searching of the N-terminal sequence (residues 1-110) of the PUL by NCBI protein blast tool with the database of PDB. The picture was generated from the webpage of http://blast.ncbi.nlm.nih.gov/Blast.cgi.

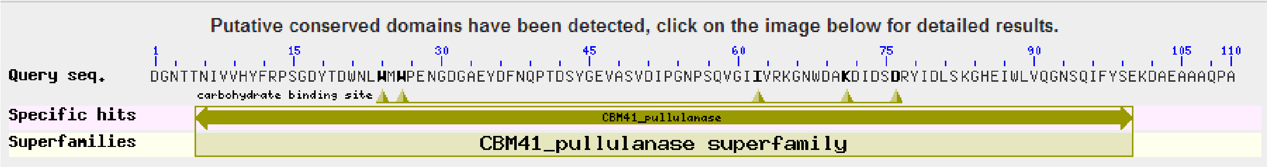

References

1. Huang, Y. J., Acton, T. B. & Montelione, G. T. in *Structural Genomics,* 3-16 (Springer, 2014).
